# Efficacy assessment for low-level laser therapy in the treatment of androgenetic alopecia: a real-world study on 1383 patients

**DOI:** 10.1007/s10103-022-03520-4

**Published:** 2022-02-08

**Authors:** Jun Qiu, Yanhua Yi, Linlang Jiang, Yong Miao, James Jia, Jian Zou, Zhiqi Hu

**Affiliations:** 1 Department of Plastic and Aesthetic Surgery, Nanfang Hospital of Southern Medical University, 1838 North Guangzhou AV, Guangzhou, 510515 Guangdong China; 2 Department of Burn, Would Repair Surgery and Plastic Surgery, Department of Aesthetic Surgery, Affiliated Hospital of Guilin Medical University, Guilin, 541001 Guangxi China; 3 Department of Plastic and Aesthetic Surgery, Affiliated Hospital of Xiangnan University, Chenzhou, China; 4 Research Department, Slinph Technologies Co., Ltd, Room 1703, Block A, Bairuida Building, 4001 Ban Xue Gang Avenue, Long Gang District Shenzhen, China

**Keywords:** Low-level laser therapy (LLLT), Efficacy assessment, Androgenetic alopecia (AGA), Real-world study (RWS)

## Abstract

Low-level laser therapy (LLLT) has been a treatment modality by many androgenetic alopecia (AGA) patients in recent years. It remained unclear as to how long the treatment regime should be maintained, and which characteristics of patients should this be recommended. A real-world study was carried out with an FDA-cleared low-level laser helmet for 1383 patients. Ordinal logistic regression analysis with propensity score matching (PSM) was used to investigate the factors related to efficacy assessment. More than 80% of users were between 18 and 40 years old. The median use times were 133 for mild AGA patients and 142 for moderate-to-severe AGA patients, which equated to 38 weeks and 40 weeks, respectively. The overall clinical effectiveness was nearly 80%. PSM analysis revealed that gender (*P* = 0.002), use period (*P* = 0.068), scalp conditions with dandruff, rash, and itchy symptoms were associated with the grading of efficacy assessment. Male users (ordinal OR: 1.35, CI: (1.01, 1.79)); use for more than 180 times or use period for 1 year (ordinal OR: 1.40, CI: (1.11, 1.96)); and those with scalp dandruff (ordinal OR: 1.34, CI: (1.01, 1.87)), rash (ordinal OR: 1.47, CI: (1.04, 2.07)), and itchy symptoms (ordinal OR: 1.51, CI: (1.12, 2.03)) had better efficacy assessments. The recommended treatment regime with low-level laser helmet was more than 1 year or 180 use times. Male patients with dandruff, rash, and itchy symptoms in scalps tended to have a better efficacy assessment.

## Background

Androgenetic alopecia (AGA) is the most common type of nonscarring progressive hair loss, characterized by gradual follicular miniaturization presenting a receding frontal hairline or notable hair loss around the vertex in men, and a diffuse thinning of the hair on the crown in women [1, 2]. Although the prevalence of AGA in Chinese men and women is comparatively lower than that in Caucasians, there is an evident tendency that prevalence of AGA has been gradually increasing with early onset in recent years [3, 4]. AGA has psychosocial distressing impact on alopecia patients that diminish their self-confidence especially among those young with more severe balding [5, 6]. Currently, the common treatment options for AGA include hair transplantation, oral finasteride, topical minoxidil. In addition, there are some additional treatments, such as injection of platelet-rich plasma (PRP) and low-level laser therapy (LLLT) [7–9].

In recent years, LLLT has been a popular treatment option by many AGA patients in China [10]. At present, there are several commercially available devices designed for home use in China, among which, the iHelmet (Slinph Technologies Co., Ltd, Shenzhen, China), an FDA-cleared (K162782) helmet-like device with lasers and light-emitting diodes, has been promoted to several thousands of users in the past 5 years.

Several previous studies suggested that low-level laser treatment would be an effective option to treat pattern hair loss in both men and women [11–14]. There were some limitations in these studies: (1) small sampling (2–225 subjects); (2) short-term study with maximum period of 26 weeks; (3) selection bias. Real-world studies use information from real clinical setting and seek to provide evidence complementary to that provided by randomized controlled trials (RCTs). To fill in the above research gap, this study aimed to determine the efficacy assessment of LLLT for 1383 Chinese AGA patients (median treated period with 38 weeks) with a real-world study using propensity score matching (PSM)analysis.

## Methods

### LLLT device and data acquisition

The iHelmet is an LLLT device with light sources consisting of two hundred laser diodes (5 mW, 650 nm) in seven scalp sections. The lasers for each device were identical in power output, and the treatment frequency was 20 min every other day. All the information including baseline data and treatment data were acquired by an APP named “iHelmet,” which could be downloaded through “Google Play” or “App Store”.

The independent variables included age groups, gender, marital status, family history with AGA, use duration, combined with medical treatment record, and scalp conditions. The severity of AGA was graded by participants first, and double-checked by two dermatologists later with the upgraded pictures according to basic and specific (BASP) classifications. The severity was dichotomized as mild (L, M0, C0, M1, C1, F1, V1) and moderate-to-severe groups (M2, C2, F2, V2, and M3, C3, F3, V3, U1∼3) [15]. The protocol was approved by the Ethics Committee of Southern Medical University, China.

### Treatment regime and efficacy assessment

There were three treatment options in this research including LLLT monotherapy, LLLT concomitant with topical minoxidil, and LLLT concomitant with oral finasteride.

The efficacy was assessed with six items: (1) whether the scalp oil secretion reduced? (2) whether the scalp dandruff reduced? (3) whether the scalp rash reduced? (4) whether the daily hair loss reduced? (5) whether new hair regrown or hair density increased? (6) whether hair changed into thicker? For the above six questions, 1-point score was given if the answer was “yes,” and a zero-score given for those questions if the answer was “no.” The overall efficacy assessment was graded by summarizing the total score for six items, “not effective” for that total score was zero, “moderately effective” for those scored 1 to 3, and “significantly effective” for those scored 4 to 6. The efficacy was assessed by participants first, double-checked by doctors with trichoscopy.

### Statistical analysis

R software (R language version 3.5.2) and EpiDisplay Package (version 3.5.0.1) were used in data analysis. Frequencies and percentages were used for descriptive variables. The differences between qualitative variables were assessed with the chi-square test or Fisher’s exact test. To reduce the selection bias conferred by potential confounding factors, a propensity score matching (PSM) analysis was performed with age, gender, and marital status matched between the mild and moderate-to-severe groups. The logit of the propensity score was nearest-neighbor matched in a 1:1 manner. Ordinal logistic regression analyses were used to model the association between the grading of efficacy assessment, demographic factors, and treatment regime. All values were two-tailed, and a *p*-value < 0.05 was considered to indicate statistical significance.

## Results

**Fig. 1:**
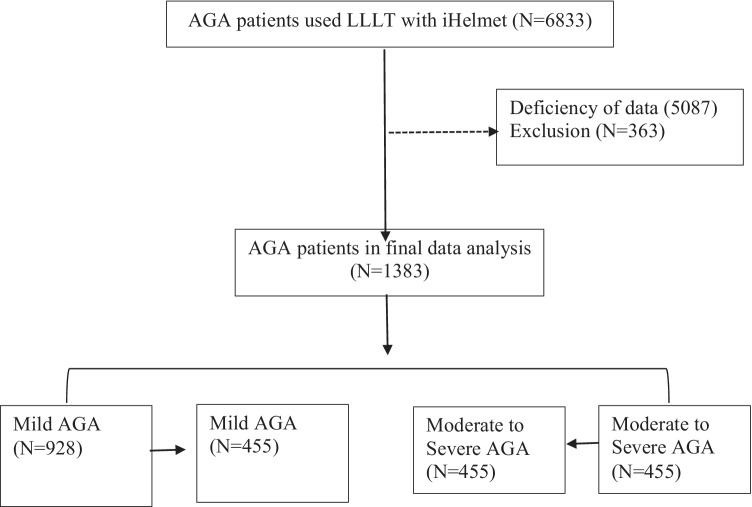
Flow chart for patents with propensity score matching

In total, 6833 AGA patients used LLLT with iHelmet; 1746 of them submitted complete information during the treatment. Three hundred sixty-three were excluded for age < 18 or age > 60; 1383 of them were included in the final analysis. To reduce the selection bias conferred by age, gender, and marital status, a PSM was performed with age, gender, and marital status matched between the mild (*N* = 455) and moderate-to-severe groups (*N* = 455), as shown in Fig. 1.

As shown in Table 1, the distribution of AGA patients using LLLT helmet in mild and moderate-to-severe groups was similar (*P* = 0.335); the majority of users (more than 80%) were between 18 and 40 years old. However, the gender difference existed (*P* = 0.007), more than two-thirds of users were male, and females in moderate-to-severe groups were more likely to use LLLT helmet whereas less likely in the mild groups. The median use times were 133 for mild AGA patients and 142 for moderate-to-severe AGA patients, which equated to 38 weeks and 40 weeks, respectively. Almost 60% of them had a family history of AGA, and more than half of them were single. Over 80% of the users presented with oil scalp. Generally, the overall efficacy assessment concluded that treatment with LLLT helmet was moderately effective for 51.9% of users with mild AGA and 57.4% with moderate-to-severe AGA, significantly effective for 27.7% users with mild AGA and 20.0% with moderate-to-severe AGA. The overall clinical effectiveness was nearly 80% by summing up the rate for moderately effective and significantly effective.

**Table 1:** Demographic data and overall efficacy assessment by 1383 alopecia patients using LLLT helmet

	Mild (*n*** = **928)	Moderate to severe (*n* = 4 55)	*P *value
Age groups, years			
18–30	451 (48.6)	199 (43.7)	0.335
30, 40	352 (37.9)	183 (40.2)	
40, 50	105 (11.3)	62 (13.6)	
> 50	20 (2.2)	11 (2.4)	
Gender			
Male	699 (75.3)	311 (68.4)	0.007
Female	229 (24.7)	144 (31.6)	
Use period, times			
Median (IQR)	133 (88,206.2)	142 (94,224)	0.017
Treatment regime			
LLLT only	591 (63.7)	287 (63.1)	0.328
LLLT and minoxidil	287 (30.9)	17 (3.7)	
LLLT and finasteride	50 (5.4)	151 (33.2)	
Family history with AGA			
No	390 (42)	178 (39.1)	0.33
Yes	538 (58)	277 (60.9)	
Marital status			
Single	458 (49.4)	202 (44.4)	0.094
Married	470 (50.6)	253 (55.6)	
Scalp conditions			
Oily scalp			
No	148 (15.9)	66 (14.5)	0.537
Yes	780 (84.1)	389 (85.5)	
Dandruff			
No	672 (72.4)	358 (78.7)	0.014
Yes	256 (27.6)	97 (21.3)	
Rash			
No	719 (77.5)	363 (79.8)	0.365
Yes	209 (22.5)	92 (20.2)	
Itchy scalp			
No	577 (62.2)	303 (66.6)	0.122
Yes	351 (37.8)	152 (33.4)	
Overall efficacy assessment			
No effective	189 (20.4)	103 (22.6)	0.008
Moderately effective	482 (51.9)	261 (57.4)	
Significantly effective	257 (27.7)	91 (20)	

Numbers in bracket are percent unless otherwise stated

After PSM by age, gender, and marital status, a PSM cohort consisted of 455 users with mild AGA and 455 with moderate-to-severe AGA are analyzed in Table 2. As shown in this table, the factors including age groups (*P* = 0.948), family history with AGA (*P* = 0.457), marital status (*P* = 0.747), treatment regime (*P* = 0.402), and oil scalp (*P* = 0.438) were not associated with the grading of efficacy assessments, while the other factors such as gender (*P* = 0.002), use period (*P* = 0.068), scalp conditions with dandruff, rash, and itchy symptoms were associated with the grading of efficacy assessment.

**Table 2:** Efficacy assessment by demographic data, scalp conditions, and treatment period in PSM cohort

	Not effective	Moderately effective	Significantly effective	*P* value
Total	198 (21.8)	510 (56.0)	202 (22.2)	
Age groups, years				
18–30	87 (43.9)	226 (44.3)	84 (41.6)	0.948
30, 40	80 (40.4)	198 (38.8)	87 (43.1)	
40, 50	25 (12.6)	73 (14.3)	27 (13.4)	
> 50	6 (3)	13 (2.5)	4 (2)	
Gender				
Female	63 (31.8)	181 (35.5)	44 (21.8)	0.002
Male	135 (68.2)	329 (64.5)	158 (78.2)	
Use period, times				
Median (IQR)	131.5 (89,206.8)	144.5 (94,224)	152 (96,241.8)	0.068
Treatment regime				
LLLT only	139 (70.2)	326 (64.2)	132 (65)	0.402
LLLT and minoxidil	56 (28.3)	162 (31.8)	63 (31)	
LLLT and finasteride	3 (1.5)	20 (3.9)	9(3.9)	
Family history with AGA				
No	88 (44.4)	205 (40.2)	78 (38.6)	0.457
Yes	110 (55.6)	305 (59.8)	124 (61.4)	
Marital status				
Single	86 (43.4)	228 (44.7)	84 (41.6)	0.747
Married	112 (56.6)	282 (55.3)	118 (58.4)	
Scalp conditions				
Oily scalp				
No	37 (18.7)	78 (15.3)	29 (14.4)	0.438
Yes	161 (81.3)	432 (84.7)	173 (85.6)	
Dandruff				
No	167 (84.3)	403 (79)	149 (73.8)	0.034
Yes	31 (15.7)	107 (21)	53 (26.2)	
Rash				
No	168 (84.8)	407 (79.8)	143 (70.8)	0.002
Yes	30 (15.2)	103 (20.2)	59 (29.2)	
Itch scalp				
No	150 (75.8)	341 (66.9)	111 (55)	< 0.001
Yes	48 (24.2)	169 (33.1)	91 (45)	

Numbers in bracket are percent unless otherwise stated

To further analyze the factors associated with the efficacy assessments, an ordinal logistic regression analysis is summarized in Table 3. Set the grading “not effective” as reference; the factors of male (*P* = 0.02), use period of more than 180 times (*P* = 0.03), scalp condition with dandruff (*P* = 0.04), rash (*P* = 0.01), and itchy symptoms (*P* = 0.003) were positively associated with efficacy assessments. It was likely that the male users (ordinal OR: 1.35, CI: (1.01, 1.79)); use for more than 180 times or use period for more than 1 year (ordinal OR: 1.40, CI: (1.11, 1.96)); and those with scalp dandruff (ordinal OR: 1.34, CI: (1.01, 1.87)), rash (ordinal OR: 1.47, CI: (1.04, 2.07)), and itchy symptoms (ordinal OR: 1.51, CI: (1.12, 2.03)) had better efficacy assessments.

**Table 3:** Ordinal logistic regression analysis of factors that associated with the efficacy assessments with not effective as a reference

Variables	Explanation of variables	Ordinal odds ratio (95% CI)	*P* value
Age, years	18–30	1.00 (reference)	
	31–40	1.02 (0.74,1.42)	0.45
	41–50	1.08 (0.69,1.69)	0.37
	> 50	0.75 (0.32,1.76)	0.25
Gender	Female	1.00 (reference)	0.02
	Male	1.35 (1.01, 1.79)	
Marital status	Single	1.00 (reference)	0.18
	Married	1.16 (0.84,1.60)	
Family history with AGA	No	1.00 (reference)	0.26
	Yes	1.09 (0.84,1.41)	
Use period, times	< 90 (half year)	1 (reference)	
	90–180 (half year to 1 year)	1.20 (0.86,1.67)	0.14
	180–360 (> 1 year)	1.40 (1.11,1.96)	0.03
Treatment regime	LLLT only	1.00 (reference)	
	LLLT and minoxidil	1.17 (0.89, 1.54)	0.14
	LLLT and finasteride	1.08 (0.55, 2.12)	0.42
Scalp conditions			
Oily scalp	No	1.00 (reference)	0.32
	Yes	0.92 (0.64,1.32)	
Dandruff	No	1.00 (reference)	0.04
	Yes	1.34 (1.01,1.87)	
Rash	No	1.00 (reference)	0.01
	Yes	1.47 (1.04,2.07)	
Itchy scalp	No	1.00 (reference)	0.003
	Yes	1.51 (1.12,2.03)	

Ordinal logistic regression analysis; *CI*, confidence interval

## Discussion

Although Endre Mester firstly documented that accelerated hair regrowth in shaved mice after exposure to a low-power 694-nm ruby laser in 1967 [16], which discovered the potential of low-level laser therapy for hair loss, it was not until in the year of 2009 that the first RCT on the role of LLLT in AGA was carried out for Hair Max Laser Comb to receive FDA clearance [17]. In the past decade, several other studies confirmed the effectiveness of LLLT on increasing hair count and hair thickness. However, these studies had some limitations. Firstly, the study enrolled few patients, and the treatment periods with LLLT were comparatively short. Secondly, these researches had not taken the scalp conditions into study. Above all, most of these studies had strict inclusion and exclusion criteria, which meant that trial populations were not good representative of the real alopecia population in real-world practice. By far, there was not any real-world study with larger sample size.

As a typical real-world study, this study filled in the above research gaps. The iHelmet made by Slinph Technology Co., Ltd, Shenzhen, China, was an FDA-cleared (K162782) helmet-like LLLT device. All data for several thousand users were extracted from the APPs linked to iHelmet the in the past 5 years. The median treatment periods were equal to 38 weeks and 40 weeks for mild and moderate-to-severe AGA patients. The study adopted a comprehensive efficacy assessment including six items. The overall efficacy assessment concluded that treatment with LLLT helmet were moderately effective for 51.9% of users with mild AGA and 57.4% with moderate-to-severe AGA, significantly effective for 27.7% and 20.0% of them, respectively; the results were better than the self-assessment of efficacy in the research conducted by Jimenez et al. in 2014 [11]. In previous studies, LLLT with a topical combination of 0.25% finasteride and 3% minoxidil improved efficacy in the treatment of FPHL with an additional benefit of increasing hair diameter [18]. Combined treatment was better in reducing oil secretion, improving hair diameter and hair density [19]. No synergistic effects were found in efficacy assessments among LLLT monotherapy, LLLT concomitant with minoxidil, and LLLT concomitant with finasteride in this study, which were different from previous research results [20].

Meanwhile, this study answered the questions on what characteristics of AGA patients should be recommended to LLLT, how long would it take for LLLT to get prime treatment effects. This study proved that male users; use for more than 180 times or use period for 1 year; and those with dandruff, rash, and itchy symptoms in scalps were more likely to have better efficacy assessments. The findings had important significance in clinical setting.

In conclusion, this was a real-world study on LLLT in the treatment of AGA with a big sample size. The overall clinical effectiveness with iHelmet for AGA was nearly to 80%. The recommended treatment regime with low-level laser helmet was more than 1 year or 180 use times. Male patients with dandruff, rash, and itchy symptoms in scalps tended to have a better efficacy assessment.
